# Conflict-induced household-level food insecurity in conflict-affected areas of Northeastern Ethiopia

**DOI:** 10.29219/fnr.v69.11736

**Published:** 2025-05-02

**Authors:** Dawit Bezabih, Mehretie Belay, Simachew Bantigegn

**Affiliations:** Department of Geography and Environmental Studies, Bahir Dar University, Bahir Dar, Ethiopia

**Keywords:** conflict, food insecurity, food consumption score, household food insecurity access scale, households, livelihood zones, Ethiopia

## Abstract

Conflict-induced food insecurity has been currently emerging to be a widespread challenge to the decent livelihood of the human population. This study examined conflict-induced food insecurity in conflict-affected areas of the northeastern part of Ethiopia. This study assessed three time periods (pre-conflict, conflict, and post-conflict times) to analyze the impact of conflict on the studied households. Food consumption score and household food insecurity access scale tools were used to measure the food security status of households. Descriptive statistics and independent t-test were used to analyze the data. The major finding confirmed that the food security status of both urban and rural households in the study areas was negatively affected by the conflict. Compared to the pre-conflict period (22.2%), the number of food-insecure households at the time of the conflict was three times higher. Though the food security status of both rural and urban households was affected by the conflict in the area, the effect was much severe for the rural households. The number of food-insecure rural households during the conflict was three times higher than the pre-conflict period. During the conflict, female-headed households (78.3%) were more vulnerable to food insecurity than male-headed households. The independent t-test result confirmed the presence of a difference in food security status between rural and urban households (*P* > 0.01) and between female- and male-headed households (*P* > 0.021). Food security status variations were also seen among the study livelihood zones. Households from the north wello east plain livelihood zone suffered a lot (71.3%). The result suggested that any project aiming at improving households’ food security in conflict-affected areas should give attention to the provision of food aid, agricultural inputs, credit services, and financial support to the affected community. Restoring peace would rather be the long-lasting solution to minimize the conflict-induced food insecurity in the area.

## Popular scientific summary

- Prolonged conflict has disrupted the livelihood strategy and outcomes of the household in the area and significantly reduced access to essential food supplies.
- Both rural and female headed households were found to be disproportionately vulnerable to food insecurity in the conflict situation.
- Urgent need for conflict resolution strategies, targeted humanitarian intervention, and sustainable recovery programs are essential to address the food insecurity problem in the conflict situation.

Food insecurity was one of the developmental challenges of the world. Nowadays, it continues to remain the key developmental challenges (1). One of the main goals, attaining the Zero Hunger target by 2030, is vastly confronted by the growing number of food-insecure people from year to year. Approximately 733 million people worldwide faced hunger, equating to one in 11 individuals. In Africa, the situation is even more sever, with one in five people experiencing hunger (2). The recent persistent global challenge, food insecurity, is attributed to many factors. Armed conflict, climate erraticism and extremes, and economic downturns are among the factors. These factors are identified as driving forces for the emergence and continuation of food insecurity at global levels (3).

Recently, the impact of conflict on food security has been steadily rising throughout the world. Conflict-induced food insecurity has been now emerging to be a widespread challenge to the decent livelihood of human population at the global and local levels. Because of the frequent rise of armed clashes, wars, disputes, and terrorism, here and there, the number of food-insecure people has been rising over the different corners of the world (3). The recently recorded food insecurity problems everywhere are likewise believed to be the direct results of conflicts. Various studies confirm that conflict is intensifying food insecurity by affecting the dimensions of food availability in one way or another. FAO et al., for instance, noted that conflict was the key reason for the rise of food-insecure and hungry people globally in 2020 and 2021 (1). Brinkman and Hendrix as well as Martin-Shields et al. added that conflicts can negatively affect the access, availability, utilization, and stability dimensions of food security (4, 5). Currently, the vilest food crises have occurred in areas of armed conflicts illuminating a strong connection between conflict and food insecurity (6). For instance, about 50% of the people in Syria and Yemen are suffering from food insecurity (7). All the 19 countries categorized by FAO as under ‘protracted crisis’ conditions in 2017 were involved in violent conflict at that time too (8). Furthermore, six out of 10 countries with severe food crisis in the world in 2019 and all the countries faced famine in 2020 were caused due to armed conflict (9).

Conflict enforces people to fall into food insecurity traps and make them impotent to bounce-back from the shocks (10). Conflict can have an overwhelming influence on a community’s food security. Armed conflicts directly cause food insecurity and starvation by disturbing markets and supply chains, killing livestock and burning of fields, or when the chief producers of food are killed or involved in battle, destroying infrastructure, increasing food price, or making goods and services unavailable altogether. Destruction to water and sanitation infrastructure due to warfare can make it more challenging to safely prepare food and highly impacts nutrition outcomes. It can be even more challenging to endure proper nutrition and resilience to food insecurity when households’ security is compromised.

Consistent to the foregoing texts, the conflict in Ethiopia believed to affect the food security status of the households. The armed conflict in northern Ethiopia was started in November 2020. A military attack against Tigray was ordered in response to the attack on federal army camps by the so-called Tigray Defense Forces (TDF), marking the start of the civil war in the area (11). Since November 2020, the conflict in the northeastern part of Ethiopia has made an intense negative impact (12). The armed conflict has led to death of civilians, mass displacement of residents, demolition of infrastructures, interruption of government services, such as banking, communication, health, education, and transport and triggered the collapse of economic activities in the northeastern part of Ethiopia (11). Crops and animals were deliberately and accidentally looted, burned, or destroyed during the conflict. As a result, most of the rural households were left without any food and agricultural inputs, which, in turn, greatly affected the primary livelihood strategy (crop production and animal rearing) of the majority of the households.

Various studies (13–15) were conducted regarding food insecurity in Ethiopia in general and in the study area in particular. Although food insecurity is attributed to both natural and manmade factors, almost all the aforementioned studies in Ethiopia assessed food insecurity from the angles of natural hazards.

Hence, studies on the level of the armed conflict linked to household food insecurity, and its outcomes in the conflict areas of northeastern Ethiopia are scarce and missing in the reviewed studies. Therefore, this study provides an important addition to the literature regarding the level of food insecurity in the conflict context. Additionally, studies on the level of food insecurity and its outcomes in the context of conflict can facilitate the identification of important strategies to be implemented by the government and communities for the improvement of food security in the area. Thus, this study has practical significance for designing more targeted and effective food security-related development interventions in the study area. The aim of this study is to analyze household-level conflict-induced food insecurity in the conflict areas of northeastern Ethiopia, particularly in Amhara regional state (focusing on the Northern Ethiopian conflict of 2020–2023).

## Materials and methods

### Description of the study area

**Fig. 1:**
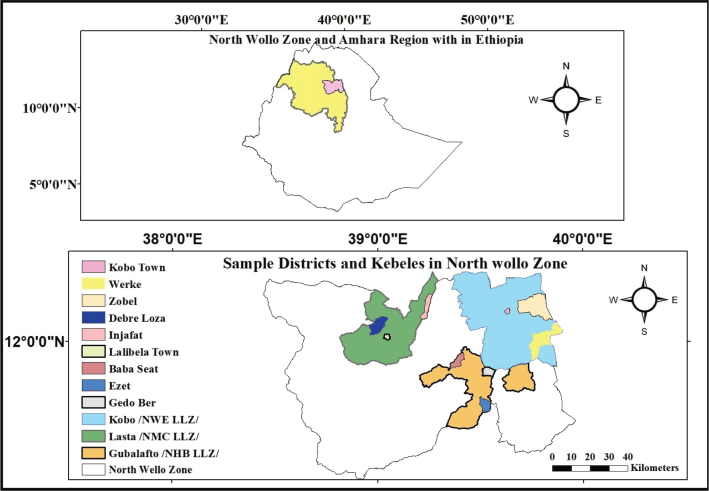
Location map of the study area in the Amhara Region, Ethiopia.

This study was carried out in the North Wollo Zone of the Amhara National Regional State (ANRS) in Ethiopia (Fig. 1). This region has been significantly affected by prolonged conflict in Ethiopia. Geographically, it is situated between 11°30' and 12°30'N latitude and 38°30' and 40°E longitude. North Wollo is characterized by a rugged and uneven topography. The area typically experiences two distinct rainfall seasons, and a large portion of the local population practices, a bimodal farming system. Agriculture serves as the primary livelihood for the majority of residents, with nearly all individuals engaged in mixed crop-livestock farming. Key crops grown in the region include teff (*Eragrostis tef*), barley (*Hordeum vulgare*), wheat (*Triticum*), sorghum (*Sorghum bicolor*), and maize (*Zea mays*). Livestock in the area comprises cattle, pack animals, sheep, goats, chickens, and, in the Kolla (lowland) areas, camels (16).

### Data and methods

A mixed-method research approach was employed, integrating both quantitative and qualitative data generation and analysis. This approach was chosen to cross-validate the findings, with qualitative data specifically used to explain unexpected results from the quantitative data. This study adopted a convergent mixed-method research design. A multi-stage sampling design and procedure were implemented to select the sample sites and households.

In the first stage, the North Wollo Zone was purposively selected due to the prolonged conflict in the area, which was believed to have had a significant impact. The selected zone was then divided into three dominant livelihood zone groups. From these, the Kobo district (North Wollo East Plain Livelihood Zone, NWE) (17), the Gubalafto district (North Wollo Highland Belg Livelihood Zone, NHB), and the Lasta district (Northeast Midland Mixed Cereal Livelihood Zone, NMC) were randomly selected. The rationale behind this selection was that households within similar livelihood zones are likely to share comparable livelihood opportunities and constraints. At the kebele level (the smallest administrative unit in the country’s governance structure), a stratified sampling approach was used. All kebeles in the three selected districts were first stratified into rural and urban categories. Following this, two rural kebeles and one urban kebele were randomly selected from each district, ensuring proportional representation (see Table 1).

**Table 1:** Sample household distribution in the study districts

Sample districts	LLZ	Sample kebeles		Number of rural HH			Number of urban HH			Total HH			Total sample HH		
		Rural	Urban	M	F	T	M	F	T	M	F	T	U	R	T
Kobo	NWE	Worke Zoble	Kobo 01	1,403	327	1,730	1,703	216	1,919	4,713	757	5,470	59	108	167
				1,587	234	1,821									
Gubalafto	NHB	Babasat Ezet	Gedober	942	181	1,123	1,005	206	1,211	2,758	586	3,344	37	65	102
				811	199	1,010									
Lasta	NMC	Debrloza Enjafat	Lalibela 02	776	232	1,008	1,423	471	1,894	2,991	897	3,888	58	61	119
				792	194	986									
Total				6,331	1,347	7,678	4,131	893	5,024	10,462	2,240	12,702	154	234	388

Note: HH: Household; LLZ: Livelihood Zone; M: Male; F: Female; T: Total; U: Urban; R: Rural; NWE: North Wollo East plain livelihood zone; NHB: North Wollo Highland Belg livelihood zone; NMC: Northeast Mid land mixed Cereal livelihood zone.

Finally, a total of 388 households were sampled (Table 1) for a questionnaire survey from the six *kebele* administrations using a proportional random sampling technique based on the sampling frames obtained from each *kebele* administration office. In this case, a simple random lottery method was employed to select the individual household as a sampling unit.

A series of nine Focus Group Discussions (FGDs) were organized, each comprising eight participants who were carefully chosen for their knowledge and experience in conflict-related matters to ensure meaningful input. The participants were three conflict victims (injured or lost family members, lost their assets), two religious leaders, one health officer, one teacher, and one kebelle administrative officer. Six of these discussions took place in rural areas, while the remaining three were held in urban locations to capture a wide range of perspectives. Each FGD was facilitated by two trained moderators responsible for steering the conversation, encouraging participation, and managing group interactions. These facilitators completed an extensive training program that covered FGD moderation, group dynamics management, and the use of probing questions to extract detailed information.

Each discussion lasted around 90 min and followed a semi-structured approach, with probing questions used to delve deeper into the topics. All sessions were conducted in Amharic, the native language of the participants. The audio recordings were transcribed verbatim in Amharic and later translated into English by professional translators. To maintain accuracy and cultural sensitivity, the research team reviewed the translations.

A team of three experienced coders, skilled in qualitative data analysis, handled the coding and analysis of the FGD transcripts. They underwent specialized training in thematic analysis, which included data familiarization, initial code generation, and theme identification. The transcripts were analyzed using thematic coding to identify recurring terms and phrases related to participants’ experiences with food insecurity and their coping mechanisms.

In addition to the FGDs, Key Informant Interviews (KIIs) were conducted to provide further insights into the study. Fifteen key informants were selected based on their expertise, professional roles, and relevance to the research goals. These informants included experts from non-governmental humanitarian organizations, risk prevention and food security specialists at district and zone levels, and local administrators and religious leaders.

The KIIs followed a semi-structured format, guided by open-ended questions aimed at gathering detailed, context-specific information. Each interview lasted between 35 and 50 min and was conducted in Amharic. With the informants’ consent, interviews were audio-recorded and later transcribed. The transcripts were translated into English by professional translators and reviewed by the research team for accuracy and cultural relevance. The KII data were analyzed thematically alongside the FGD data to identify key themes and patterns.

Informed consent was obtained from all participants involved in the FGDs and KIIs before the sessions commenced. Participants were assured of the voluntary nature of their involvement, the confidentiality of their data, and their right to withdraw from the study at any point. They were also informed that they could request the deletion of their interview recordings if desired. The data collection process received ethical approval from the Bahir Dar University Department of Geography and Environmental Studies Ethical Committee (Approval Number: ጂኦ/B21/2016, Date: December 12, 2022).

In addition to primary data collection, secondary data were gathered through web searches, including documents and reports on conflicts and food insecurity in conflict-affected regions worldwide. These materials were analyzed to provide comparative and supplementary insights. The fieldwork was carried out between April and May 2023. The questionnaire used in the study included both closed and open-ended questions and was administered with the support of nine trained enumerators, who were recruited from each of the sample districts.

To analyze the impact of the conflict on food security, the study used three time periods (2020, as a pre-conflict period; 2021–2022, as a conflict period; and the data collection time as a post-conflict period). Hence, data were gathered for three time periods through a questionnaire survey of 388 households – nine FGDs and 15 KIIs. The questionnaire was based on validated food security assessment tools, namely, the Food Consumption Score (FCS) and the Household Food Insecurity Access Scale (HFIAS). These tools are widely recognized and have been extensively used in food security research globally. To ensure reliability, enumerators were trained to administer the tools consistently, and pre-testing was conducted to adapt the tools to the cultural and contextual specifics of the study area. This included modifications for local food items and terminology while maintaining the core constructs of the tools. The themes of the questionnaire align with the objectives of assessing food security, with FCS capturing dietary diversity and food frequency, and HFIAS addressing the experiential aspects of food insecurity. To measure the food security status of the households in the study area, HFIAS and the FCS were used.

### Food consumption score

The FCS is one of the most commonly used methods of measuring household food security (18). It mainly measures the ‘access’ dimension of food security (19–21). In many ways, conflict highly affects the access dimension of food security. As a result, in order to assess the dimension of food security, applying FCS is better (21). In this study, the original FCS tool of the WFP was slightly adapted to cover the three time periods. FCS is a composite score used to capture the dietary diversity and food frequency of different kinds of food or food groups consumed during 7 days. FCS involves the weighing of these groups, given a score that represents the diversity of intake of each household (22). Households were asked about the number of days each of the food items was consumed in their homes within a week. After identifying the number of days each food was consumed, the consumption frequency of the food items is then multiplied by the corresponding food group weights.

The resultant values were then added to obtain the FCS for each household:

FCS = 2*Y staple + 3 * Y pulse + 1 *Y vege + 1 * Y fruit + 4 * Y animal + 4* Y diary + 0.5 * Y sugar + 0.5 * Y oil,

where Y represents the number of days, each food item was consumed within a week. After estimating the FCS of the sampled households, their food consumption (access) was classified into three categories according to the guidelines provided by World Food Program (18) (Table 2).

**Table 2:** Food consumption score and household category

FCSs	Household categories
Score 0–28	Poor
Score 28.5–42.5	Border line
Score >42.5	Acceptable

FCS: food consumption score.

### HFIAS score

The HFIAS is a popular tool used by the United States Agency for International Development (USAID) Food and Nutrition Technical Assistance III Project (FANTA). The tool is designed to collect information on: feelings of anxiety over food (e.g. access and resources); concern that food is insufficient in quantity and quality for children and adults (includes preference, dietary diversity, and nutritional adequacy); informing the reductions in food intake for the households (including adults and children); and feelings of embarrassment to turn to socially unacceptable means to obtain food resources. About to the guideline, Coates et al. (23) noted that the sampled households were asked 13 questions regarding their food insecurity over three time periods. Often is coded as 4, sometimes as 3, rarely as 2, and never as 1. The scores on all the questions were then added in order to get the HFIAS score for each household. The total of HFIAS scores was then categorized as: sever food insecure (27–39), moderate (14–26), and mild/no (1–13). Since there is no guideline in the literature about the categorization of HFIAS scores, this study used an equal interval method in this categorization. Finally, descriptive statistics and independent t-test were used to analyze the data. The independent sample t-test was computed to see whether there is a difference in mean of level of food security status among the rural and urban households. In this case, household’s food security status is the dependent variables, and households’ location (rural and urban) and gender of head of the household (male and female) are the independent variables.

## Results and discussion

### Sociodemographic characteristics of the respondents

The demographic profile of surveyed households reveals that most household heads (66.2%) fall within the 35–55 age range, which is considered the prime working age. A smaller proportion (15.2%) is in their twenties, while 18.6% are aged 56 or older (Table 3). In Ethiopia, male-headed households are predominant, with national data indicating that over 76% of households are led by men (CSA, 2012). This study aligns with that trend, showing that 78.1% of the surveyed households were headed by males (Table 3).

**Table 3:** Sociodemographic characteristics of respondents

No	Variables	Group	Number	Percentage
1	Age	25–35 36–45 46–55 56–65 >65	59 142 117 42 28	15.2 36 30.2 10.8 7.8
2	Sex	Male Female	303 85	78.1 21.9
3	Marital status	Married Unmarried Divorced Widowed	222 37 45 84	57.2 9.5 11.6 21.6
**4**	Educational status	Literate Illiterate	162 226	41.8 58.2

Regarding marital status, the majority (57.2%) of household heads were married. Meanwhile, 11.6% were divorced, 21.6% were widowed, and the remaining respondents had never married (Table 3). Educational attainment was notably low, with 58.2% of respondents identified as illiterate (Table 3).

The FGD included 72 participants, with a greater share (66.7%) coming from rural areas (48 individuals) and the rest (33.3%) from urban settings (24 individuals). Men accounted for 65% (47 participants), while women made up 35% (25 participants).

In terms of literacy, 48.6% (35 participants) had some level of education, whereas 51.4% (37 participants) were illiterate. Age distribution showed that a significant majority (81%) were between 35 and 55 years old. Additionally, 11% (8 participants) were between 25 and 35, while 8% (6 participants) were over 55 years old. Marital status among focus group participants reflected a similar pattern, with 74.6% (54 individuals) being married, 22% (16 individuals) widowed, and 3.4% (2 individuals) unmarried.

### Food security status of the households in the three time periods

The food security status of households was evaluated using FCS and HFIAS scores across three time periods: before the conflict, during the conflict, and at the time of data collection. As illustrated in Table 4, household FCSs showed a noticeable decline both during and after the conflict when compared to the pre-conflict period.

**Table 4:** Household food consumption score and Household Food Insecurity Access Scale computations over the three time periods

FCS level	% of FCS score			HFIAS level	% of HFIAS score		
	Before	During	After		Before	During	After
Poor	22.2	66.5	49	Severe/food insecure	30.7	79.1	63.7
Border line	52.1	28.1	38.1	Moderate	44.6	19.3	28.4
Acceptable	25.8	5.4	12.9	No/mild/secured	24.7	1.5	8.0
Total	100.0	100.0	100.0	Total	100.0	100.0	100.0

FCS: food consumption score; HFIAS: Household Food Insecurity Access Scale.

An analysis of food security categories reveals that prior to the conflict, more than half of the households (52.1%) fell within the borderline category. Meanwhile, 25.8% had an acceptable food consumption level, while 22.2% were categorized as having poor food consumption. However, during the conflict, there was a significant shift, with the proportion of households in the poor category rising sharply to 66.5%. This represents more than a threefold increase compared to the pre-conflict period, likely due to the direct impact of the conflict on food access and availability. In support of this, Weldegiargis et al. stated that armed conflict leads to loss of income and disruption of agricultural activities and production (9).

**Fig. 2:**
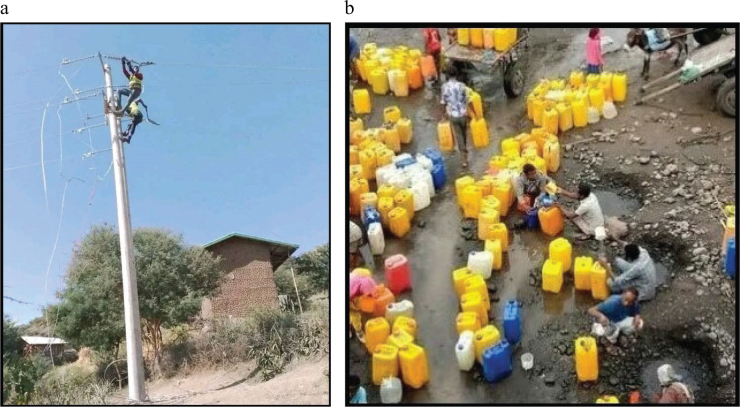
Overview of destructed infrastructures during the Northern Ethiopian conflict (2020–2023). (a) Electricity technicians maintaining the war-destructed electric lines in NWE livelihood zone. (b) Households from Gedober *Kebelle* fetching unsafe water from a nearby stream due to destruction of pipe water during the conflict. NEW: North Wollo East plain livelihood zone.

Disruption of agricultural production, off-farm income-generating activities, and loss of income, as a result of armed conflict, have primarily affected the food systems through their negative effects on people’s access to food, including the unaffordability of foods. The unaffordability of foods might be a consequence of the effects of other factors on people’s income and the cost of foods throughout the food system. The unaffordability of food together with low incomes elucidates why the households were not able to access even the cheapest food in conflict-affected districts. Key informants noted that at the time of the conflict, nothing intruded on the area. Everything was banned. Households were not able to get food items. Even, access to safe water and electricity was a major challenge at the time (see Fig. 2).

As shown in Table 4, the proportion of households within the borderline and acceptable category was low. During the time of the conflict, only 28.1 and 5.4% of the households fell under the borderline and acceptable category, respectively. Similarly, WFP’s food security assessment report on Tigray war noted that 83% of the households in Tigray were food insecure during the time of the conflict (24). After the conflict, as compared to the conflict period, there was a slight increase in the proportion of households within the acceptable category (12.9%). However, the proportion was lower when it was compared to the pre-conflict period. After the conflict, almost half (49%) of the households were under the poor category. An FAO and WFP joint report on monitoring food security in countries with conflict situation also revealed that though ongoing progresses in the food security situation was expected after the conflict, many prone groups continued to depend on humanitarian assistance (25). Households in the borderline category rose to 38.1% after the conflict compared to the 28.1% of the conflict period. Key informants noted that the slight improvement in FCS score after the conflict was due to free-market access, aid from government and non-government organizations, and support from families from non-conflict-affected areas. The key informants further noted that households in the study districts are highly in need of support to the restoration of their livelihood strategies. Hence, the supply of agricultural inputs, credit services, and financial supports are essential and will help both rural and urban households to reinstate lost materials and restart ceased business activities. Similarly, a FGD participant from NHB livelihood zone explained that:

…… the conflict completely destroyed my agricultural materials including livestock. Currently I have nothing in my hand to proceed farming. I am really in need of cash or credit to resume the agricultural activity. Just inform the government to facilitate access to credit and provide us fertilizers and other essential materials.

The distribution of the HFIAS score presented in Table 4 reveals a similar finding to that from the FCS score. The percentage of households with no/mild, moderate, and severe food insecurity at the pre-conflict time was 24.7, 44.6, and 30.7%, respectively. However, at the time of the conflict, the proportion of households in the no/mild, moderate, and severe food insecurity was correspondingly changed to 1.5, 19.3, and 79.1%, reflecting the serious impact of conflict on households’ access, quality, quantity, and affordability of food. After the conflict, there was a slight increase in the proportion of households within the mild/no (8%) and moderate (28.4%) categories, compared to that conflict period – a slight decrease within the severe category (63.7%). Lin et al. in their study of food insecurity in the context of Palestinian conflict revealed that conflict reduces the availability of production input and income, increases the number of days households have to rely on less preferred foods, and limits the variety of foods eaten and the portion size of meals consumed (26).

When the data are analyzed by Kebele type (rural vs. urban), the proportion of food-insecure households before the conflict showed only a slight difference, with 22.8% of rural households and 21.1% of urban households falling into the poor category (Table 5). An independent *t*-test (Table 6) indicated that this difference in food security status between rural and urban households was not statistically significant at that time. However, during the conflict, the gap widened considerably. The proportion of food-insecure rural households surged to 78.8%, while urban households in the poor category increased to 46.3%. Justino noted that since most conflicts are held in rural parts of a country, rural households bear the brunt of the conflict a lot (27). Verwimp likewise revealed that a large part of the production of crops occurs for own consumption for many of the African households. Hence, the disturbance of the production process and incapability to cultivate through conflict directly affects the command over food of the household members (28). A study conducted by Weldegiargis et al. also stated that most households residing in the rural part of Tigray faced a serious food security problem at the time of the conflict (9).

**Table 5:** Percentage of FCS and HFIAS categories by nature of kebeles and gender in the three time periods

Level	Percentage of rural households FCS score			Level	Percentage of rural households HFIAS score		
	Before	During	After		Before	During	After
Poor	22.8	78.8	48.5	Severe	33.6	83	65.1
Border line	53.9	16.2	39	Moderate	42.3	15.4	26.1
Acceptable	23.2	5	12.4	No/mild	24.1	1.7	8.7

Level	Percentage of urban households FCS score			Level	Percentage of urban households HFIAS score		
	Before	During	After		Before	During	After

Poor	21.1	46.3	49.7	Severe	25.9	72.8	61.2
Border line	49	47.6	36.7	Moderate	48.3	25.9	32
Acceptable	29.9	6.1	13.6	No/mild	25.9	1.4	6.8

Level	Percentage of FCS of Male-headed household			Level	Percentage of HFIAS of Male-headed household		
	Before	During	After		Before	During	After

Poor	20.1	63	46.2	Severe	28.7	77.8	62.7
Border line	52.1	31.3	40.6	Moderate	43.5	21.1	29.7
Acceptable	27.7	5.6	13.2	No/mild	27.7	0.99	7.6

Level	Percentage of FCS of Female-headed household			Level	Percentage of HFIAS of Female-headed household		
	Before	During	After		Before	During	After

Poor	29.4	78.8	58.8	Severe	37.6	83.5	67
Border line	51.7	16.4	29.4	Moderate	48.2	12.9	23.5
Acceptable	18.8	4.7	11.7	No/mild	14.1	3.5	9.4

FCS: food consumption score; HFIAS: Household Food Insecurity Access Scale.

**Table 6:** T-Test comparison of FCSs during the conflict by category of households

Household category	*N*	Mean	Std. dev	Std. error	*F*	Sig
Rural	241	1.26	0.542	0.035	18.70	0.000
Urban	147	1.60	0.604	0.050		
Male-headed	303	1.43	0.598	0.034	13.72	0.021
Female-headed	85	1.26	0.538	0.058		

FCS: food consumption score.

As to information from key informants, the major reason for the rise of food insecurity in the studied districts was the reduction of production as a result of the conflict in the area. The conflict in the area led crops and animals to be plundered, burned, or destroyed. As a result, most of the rural households left without any food and agricultural inputs. When food production, storage, and distribution processes are endangered, the supply to local markets decreases while the demand for food increases. This pushes prices to increase: more people need to depend on the market, while the market obtains low food to be sold. If the local markets can receive supplies from markets further away, the rising burden on prices may be hard. The higher the price, the more unaffordability of the households and leads them to be food insecure. An independent sample t-test result (Table 6) confirmed that during the time of the conflict, rural households were statistically significantly food insecure (1.26 ±.542) compared to urban households (1.60 ± .60) *F* = 18.7, *P* < 0.001.

The foregoing text indicates that there was significant food insecurity difference between the rural and urban as well as between male-headed and female-headed households during the time of the conflict. But, the FCS of urban households under the poor category was so high at the time of the conflict. Referring to Table 5, it is possible to note that only 6.1% of the households were food secure, 46.3% were food insecure, and the remaining 47.6% were at the borderline during the time of the conflict. Lack of physical and economic access to sufficient food because of the intense fighting and siege was among the reasons for the high levels of food insecurity in the urban study communities during the conflict time. High cost of food and persistently high levels of war-induced poverty continued to keep foods out of the reach of people in the urban areas of the study districts. The conflicting parties cut off the local communities from the supply of food and non-food items from other non-conflict areas. These conditions have aggravated the local food insecurity in the area and may potentially lead the population to famine as like reported in (29). Hence, during the conflict, urban households faced serious food supply problems.

Most of the conflicts in northeastern Ethiopia happened in rural areas. This has barred the rural households to sell their products, which, in turn, affected the food supply in the urban markets. Even after the conflict, the percentage of households who fall under the poor category was slightly higher than it was during the conflict (≈49.7%) (Table 5). As noted by key informants, the destruction of assets and livelihood resources by the armed conflict, in addition to the wobbly economic, social, and political environments, has meaningfully influenced the ability of affected households to recover their economic and social positions in post-conflict settings. In conformity to this, a joint report from FAO and WFP revealed that despite progresses in security, the food insecurity situation in most conflict-affected area continued its downhill spiral with millions of people in need of urgent food, nutrition, and livelihood assistance in 2020. Conflict-related destruction of livelihoods and disruption of agriculture, markets, and trade flows drove this deterioration, for example, in South Sudan (10).

The distribution of the HFIAS score (Table 5) shows a similar finding to that from the FCS score. According to the HFIAS score, before the onset of the conflict, 33.6 and 25.9% of the rural and urban households were severely/food insecure, respectively. However, at the time of the conflict, the number of food-insecure rural and urban households rose by 247 and 281%, respectively. A similar finding was reported by (9). In their study of food security and armed conflict in Tigray, they noted that 84.6% (by HFIAS) of households in Tigray were food insecure during the Tigray war. The augmented occurrence of household food insecurity and increment of food insecurity conditions were mainly due to the disturbed production and economic activities of the households accredited to the armed conflict. As reported by the Key informants, livestock was slaughtered and/or plundered, production highly dropped, poultry out-growers were interrupted, and milk processes were disturbed, implying that the armed conflict worsened household food insecurity by affecting the quantity, quality, and affordability of food in the study communities. After the conflict, the food security status in the study area improved. The proportion of rural and urban households that fell under the severe category was 65.1 and 61.2%, respectively.

The analysis of household FCS and HFIAS by gender reveals significant differences across different time periods. Before the conflict, the proportion of male-headed households classified as food insecure was 20.1%, while female-headed households were higher at 29.4%. However, these figures changed drastically during the conflict. The percentage of food-insecure female-headed households rose to 78.8%, nearly three times the pre-conflict level. Similarly, male-headed food-insecure households increased to 63% during the conflict (Table 5).

An independent sample *t*-test (Table 6) further confirmed that female-headed households experienced significantly higher food insecurity during the conflict (1.26 ± 0.538) compared to male-headed households (1.43 ± 0.598), with *F* = 13.72 and *P* < 0.0021. Although some improvement was observed after the conflict, food insecurity remained high for both groups, with 58.8% of female-headed and 46.2% of male-headed households still classified as food insecure. Food security analysis studies also confirmed that females are highly exposed to food insecurity. For example, FAO in its report revealed that women and girls are more food insecure and more susceptible to food insecurity than men in every province of the world (30). Similarly, a report from IPCC noted that due to gender rules and roles and growing male migration in starvation hotspot countries, food insecurity upsurges burden and duty of obtaining food on women and girls (31). Drivers of this gender gap are multifaceted and aggravated by factors, including conflict, climate change, poverty, and financial crises (32–34).

**Fig. 3:**
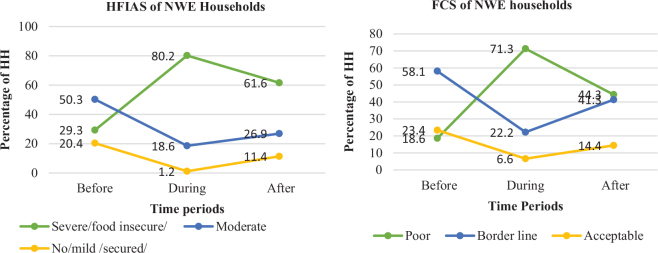
Household FCS and HFIAS computation results in the NWE livelihood zone over the three time periods. FCS: food consumption score; HFIAS: Household Food Insecurity Access Scale; NWE: North Wollo East plain livelihood zone.

When the effect of the conflict is evaluated by livelihood zones, the worst food security situation was observed in households from the NWE livelihood zone during the conflict time. According to FCS score results, during the time of the conflict, most of the households (71.3%) from the NWE livelihood zone were food insecure (Fig. 3).

With similar pattern, a large proportion of the sampled households (80.2%) were severe/food insecure with consideration of the HFIAS score results (Fig. 3). This was probably mainly because a very destructive fighting was held in the NWE livelihood zone compared to the other livelihood zones. Households in the area of under destructive conflict are severely food insecure than those households in the normal conditions. This is because the devastating nature of the conflict, the time the conflict stayed in the area, and the nature of the weapon employed in the conflict area have determined the households’ economic situation of the area.

**Fig. 4:**
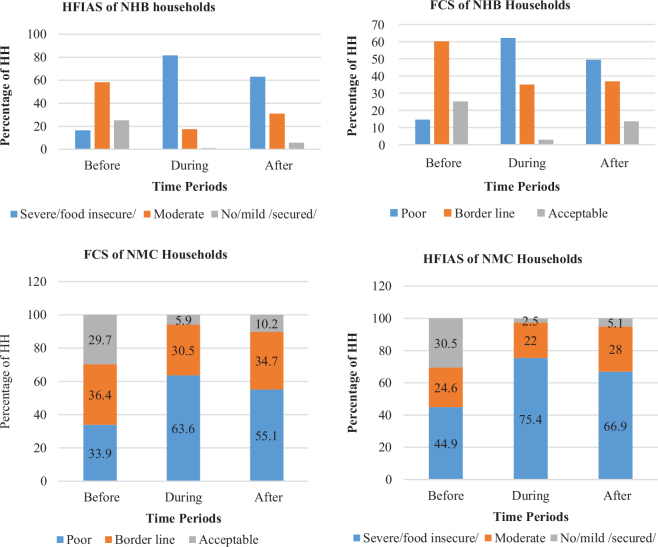
Household FCS and HFIAS computation results in the NHB and NMC livelihood zone over the three time periods. (a) Household FCS and HFIAS computation results in the NHB livelihood zone over the three time periods. (b) Household FCS and HFIAS computation results in the NMC livelihood zone over the three time periods. FCS: food consumption score; HFIAS: Household Food Insecurity Access Scale; NHB: North Wollo Highland Belg livelihood zone; NMC: Northeast Mid land mixed Cereal livelihood zone.

Households from the NHB and NMC livelihood zones equally suffered from food insecurity at the time of the conflict. As can be seen in Fig. 4a and b, 81.6 and 75.4% of the households from the NHB and NMC livelihood zones were correspondingly food insecure (with respect to HFIAS) during the conflict period. The FCS result also confirmed that 62.6 and 63.1% of the households in the foregoing respective livelihood zones experienced food insecurity during the period of the conflict (Fig. 4a and b). After the conflict, there was a slight difference in the households’ food security status over the three livelihood zones. Nearly 62, 63, and 67% of the households from the NWE, NHB, and NMC livelihood zones, respectively, were food insecure (Figs. 3 and 4).

### Comparison of food insecurity between the peace and conflict times

Household access and concern to variety, affordability, quantity, and quality of foods are furthermore analyzed for the peace and conflict times. Accordingly, households have witnessed a lessening in food quality, variety, or quantity because of laughable capitals or resources. During the time of the conflict, 82.4% of the households reported that they were concerned that enough food may not be available in the household. On the other hand, 79.7% of the households were not able to eat preferred foods. However, before the conflict, only 20.3 and 14.9% of the households were worried about the availability of enough food and were not able to eat preferred foods, respectively (Table 7). Moreover, households were obliged to eat a few kinds of foods, eat smaller meals, and skip meals at the time of the conflict. A substantial proportion of households had no food of any kind to eat (63.3%) and went a whole day and night (58.5%) without eating any food suggesting the severity of the armed conflict-induced household food insecurity. A similar finding was reported by (35). In their study of food insecurity in conflict-affected regions in Nigeria, Azad and Kaila found that 79, 71, and 74% of the households in the northern and southern parts of the country were, respectively, not able to access enough food of any kind to eat during the conflict (35).

**Table 7:** Percentage comparison of food insecurity access-related conditions between the peace and conflict times

HFIAS questions	Before the conflict (%)				During the conflict (%)			
	1	2	3	4	1	2	3	4
Have no enough food to eat	26	28.7	15.7	4.6	8	9.5	49.7	32.7
Not able to eat notorious food	43.8	17	10	4.9	5	12.6	30	49.7
Eat only few kinds of food	32.5	17	19.8	5.9	11	26.6	24.5	41.7
Skip meals	32.7	27	12	2.8	18	2	31	47
Eat less than you thought you should	40	18	13.6	3.4	2.3	15	36	43.8
Household ran out of food	35	18.8	17.5	3	2.8	23	24.5	147
No food to eat of any kind	37.6	16.5	17.5	3	5.6	22.4	28.6	34.7
Hungry but did not eat	37	20.8	14.6	2.6	4.8	23.7	35.8	34.7
Go a whole day and night without eating	41.5	23	9	1.8	33.5	4	33.5	25
Food price market was unaffordable	45	33.5	17	3.4	5.4	13.9	31.9	48
Not to find the food that you needed in the market?	87.6	11	1	0	19.8	1.3	50	27.8
Worried about the bad quality of the food	34	25	10	5.4	12.8	4.9	64.6	16.7
Unable to get food in the market due to transport	88	10.8	1	0	5	14	17.8	62

Note: 1: Never; 2: Rarely; 3: Sometimes; 4: Often. HFIAS: Household Food Insecurity Access Scale.

As learned from FGDs, households faced a serious deficiency of physical and financial access to adequate food due to the fighting and siege. The unaffordability of food and insistently high levels of poverty sustained keeping the people not to reach to the foods in the study districts. Food prices were unaffordable for 79.9% of the households at the time of the conflict (Table 7). Azad and Kaila revealed that food prices were foremost causes of food insecurity during conflict times in the aforementioned regions of Nigeria (35). One urban NHB FGD participant narrated his experience for the study area as:

Before the conflict, the price of a kilogram of teff was ETB 45. However, in 2020–2021, it rose sharply to ETB 100, and following the conflict, it further increased to ETB 135. The cost of food items has escalated significantly, with some prices doubling or even tripling. For instance, while a 5 kg sack of rice previously cost ETB 150, the current price for just one kilogram has reached ETB 110.

This practical evidence on price effects of armed conflict reported an upsurge in prices of staple food. Justino, in his study of impact of armed conflict on household welfare, stated that the demolition of roads, bridges, and other infrastructure rise the transaction costs for households involved in market exchanges and, in extreme cases, result in a return to subsistence activities (36).

### Limitation of the study

While our analysis contributes substantially to the literature and policy makers regarding conflict-induced food insecurity, it also has some limitations. One of the limitation of this study was households recall bias. Households were struggled to accurately recall and provide answers for food consumption questions, especially before and during the conflict period either because of stress or trauma. The limitation was addressed by triangulation, using complementary data sources. We also used visual aids and examples to prompt memory. Additionally, asking contextual and follow-up questions to clarify or probe for more detailed explanation ensured the consistency of the respondents and reduced vague or general answers.

## Conclusion

This study explored the conflict-induced household-level food insecurity in the conflict areas of northeastern Ethiopia. Questionnaire surveys, FGDs and KIIs, were used to collect data. FCS and HFIAS tools were employed to measure the food security status of the households in the conflict and peace times. The result confirmed that food insecurity was the major problem for both rural and urban households in all the three time periods. However, the degree of food insecurity was severe at the time of the conflict. Before the onset of the conflict, only 22.2% of the households in the area were food insecure. However, during the conflict, the number of food-insecure households was three times higher than the pre-conflict period number. Although the number of food-insecure people showed a slight decrease from the conflict period, the proportion of households under the food-insecure category after the end of the conflict was high (49%), indicating the impact of conflict on food security. The result from the HFIAS score also indicates a similar pattern. Before the conflict, 30.7% of the households in the area were severe/food insecure. However, this figure rose during the conflict period and reached 79.1%. Though the conflict affected both rural and urban households, the influence was severe on the part of the rural households. Based on the FCS and HFIAS scores, 78.8 and 83% of the rural households were food insecure, respectively. During the time of the conflict, the worst food security situation was observed in households from the NWE livelihood zone. The findings of the study imply that any project implemented by both governmental and non-governmental organizations aiming at the improvement of both rural and urban households’ food security in conflict-affected areas should give attention to the provision of food aid to the affected community, which will help them to meet their basic needs, reducing the pressure to sell of asset or engage in unsafe survival strategy. The provision of support to the restoration of the households’ livelihood strategies through the supply of agricultural inputs, credit services, and financial supports helps both rural and urban households to reinstate lost materials and restart ceased business activities. Additionally, road and market restoration rehabilitate key infrastructure, such as roads and markets to reconnect rural and urban areas enabling farmers and business owners to access market and essential services.

## Data Availability

Additional data can be accessed from the lead author (bezabih.dawit8@gmail.com) through request.
